# Buffalo Dental Association

**Published:** 1865-01

**Authors:** 


### Proceedings of Societies.

### BUFFALO DENTAL ASSOCIATION.

Minutes of meeting held Dec. 5th, 1864.

The meeting was called to order at 8 P. M. by the Presinent, Dr. Geo. E. Hayes.

The subject of the Dental Protective Union, having been made the special order for the evening, was taken up. After the reading of several documents which had been received, Dr. Whitney read a letter which he had received from the Corresponding Secretary of the Union, and spoke at length, explaining the objects aimed at by that body.

After some discussion, the following resolution was offered by Dr. Giffing, and was unanimously adopted:

"*Resolved*, That this Association recommends to its members to become members of the Dental Protective Union. Those desirous of doing so are requested to leave their names with the Secretary to be forwarded."

The regular subject for the evening: "The effect of diseased teeth upon the general health," was then taken up.

Dr. R. G. Snow enumerated quite a number of diseases which had been shown to have their origin, at times, in the irritation caused by diseased teeth, and remarked upon the neglect of physicians generally, to pay sufficient attention, or attach proper importance to diseases of the teeth.

Dr. Giffing described a case of what had every appearance of being pulmonary disease, which was cured by the extraction of the teeth.

Dr. Whitney said he considered the subject by far the most important which had yet been brought up for discussion, and expressed his regret, that not only physicians, but the greater portion of dentists did not take more pains to inform themselves upon it. He then contrasted dentistry viewed as a profession, and as a mechanical art; saying that upon the study of this subject, hinges all the difference between the true doctor of Dental Surgery, and the mechanical dentist. He then described the origin and distribution of the fifth pair of nerves; drawing attention to their intimate connection with other nervous trunks. As an instance of the effects produced by such connections, he described a case of paralysis of the right arm, induced by dental irritation. He also described a case of amaurosis arising from the same cause.

Dr. Oliver described two cases of apparent pulmonary disease caused by decayed teeth.

Dr. Southwick remarked that he had seen a similar case.

A committee consisting of Drs. Oliver, Brown and Whitcomb, were appointed to select subject and essayist for next meeting. They subsequently reported "Mechanical Dentistry," as a subject, and Dr. Geo. E. Hayes as essayist.

The meeting then adjourned.

